# Supplementary material for: Robust Validation and Comprehensive Analysis of a Novel Signature Derived from Crucial Metabolic Pathways of Pancreatic Ductal Adenocarcinoma
Source: Cancers (Basel). 2022 Apr 4;14(7):1825. doi: 10.3390/cancers14071825 (PMC8997486; doi:10.3390/cancers14071825)
Supplement: Supplementary file 1 [file cancers-14-01825-s001.zip › supplementary figures.pdf]

Supplementary Figures

Figures S1-S4

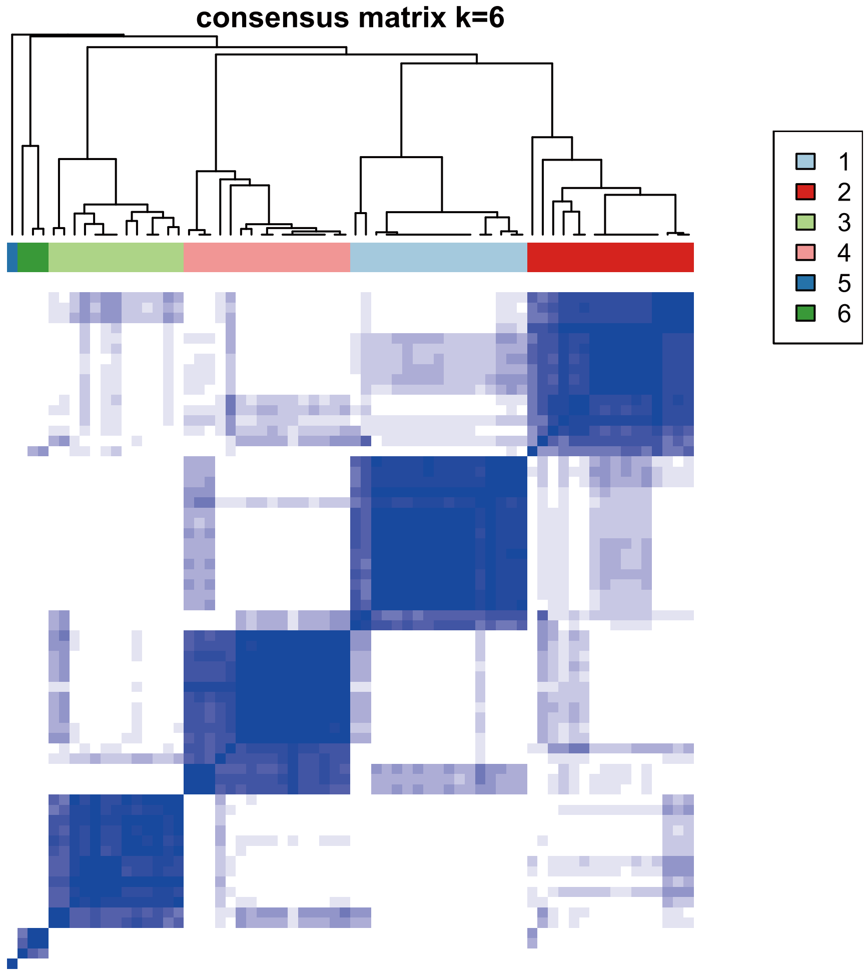

Figure S1 Consensus clustering of the ICGC cohort (n=66), k=6 was selected.

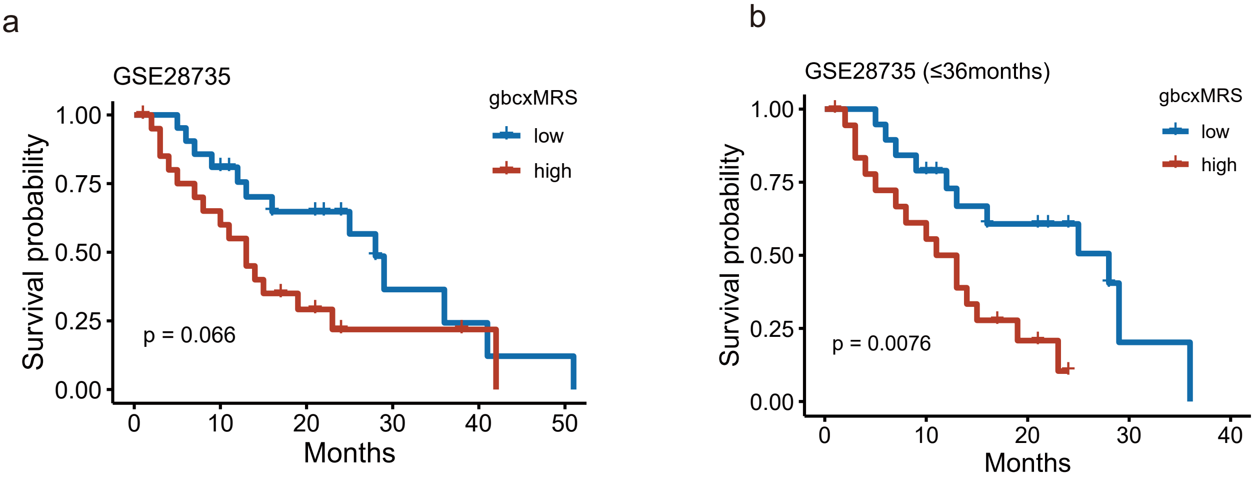

Figure S2 Validation on GSE28735. (a) All patients were included (n=42). (b) Patients with OS less than 36 months were included (n=38).

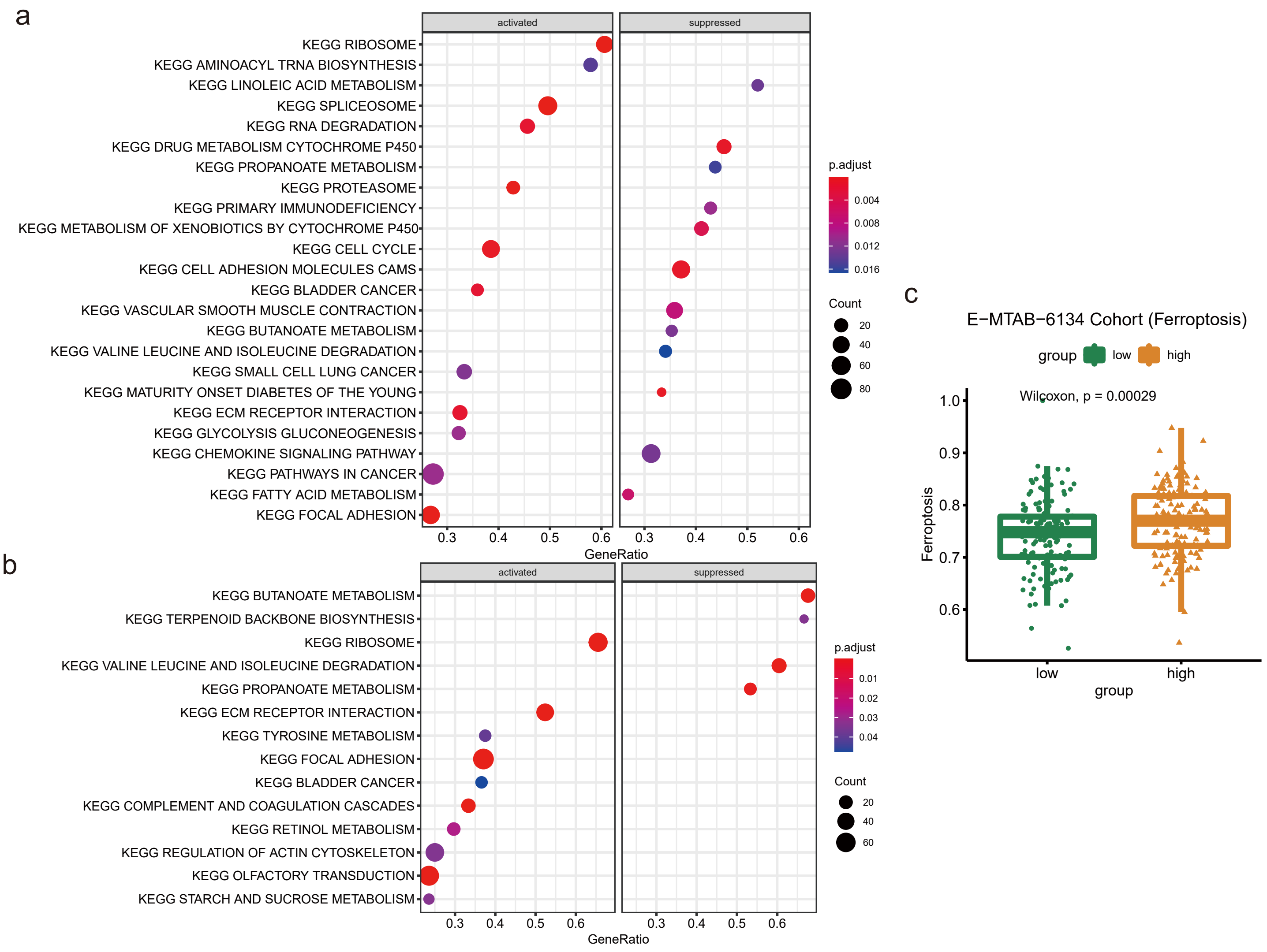

Figure S3 Functional Enrichment for gbcxMRS. (a) Gene Set Enrichment analysis (GSEA) of E-MTAB-6134 cohort. (b) GSEA of ICGC cohort. (c) ssGSEA enrichment score of ferroptosis between high and low gbcxMRS groups.

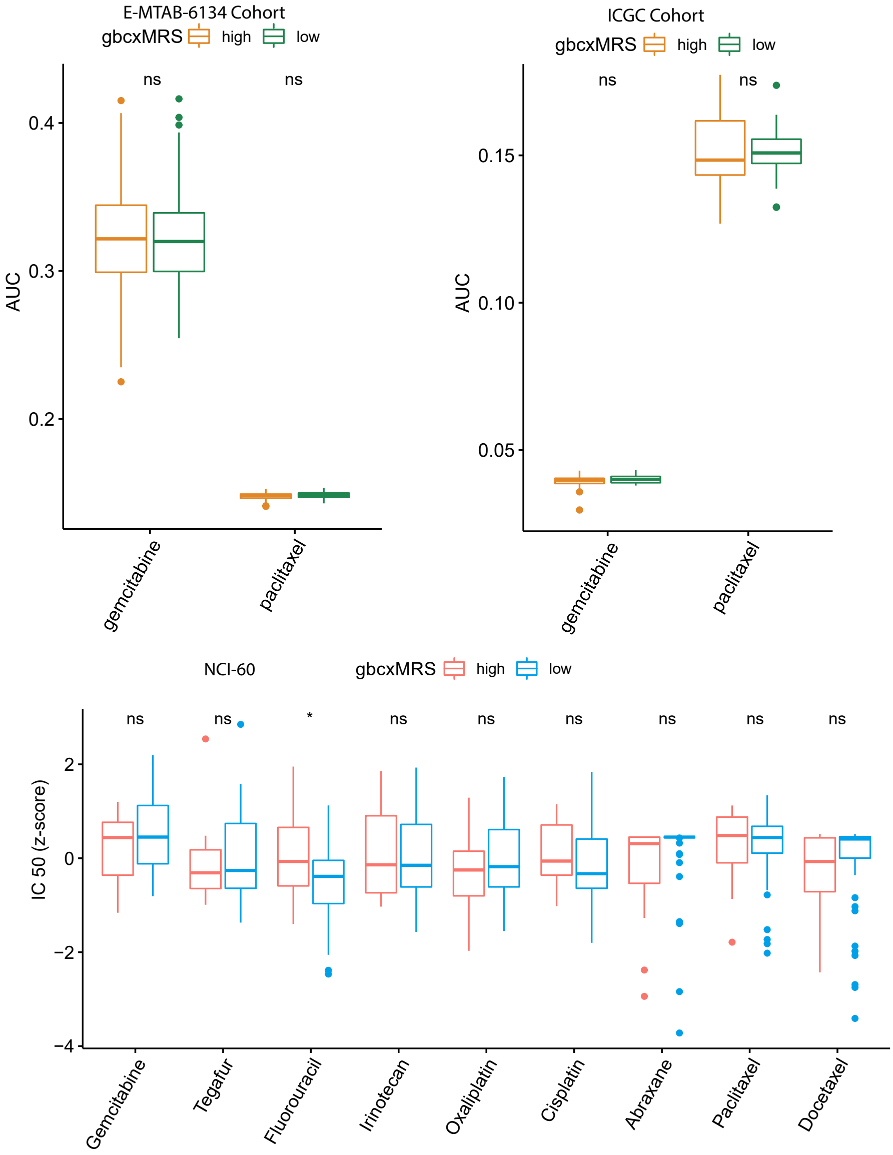

Figure S4 The drug sensitivity analysis with gbcxMRS. (a-b) There is no significant difference between gbcxMRS groups in the drug sensitivity of gemcitabine and paclitaxel. (c) The low gbcxMRS group showed the significant sensitivity of 5-fluorouracil.
